# Relationship of CD146 expression to secretion of interleukin (IL)-17, IL-22 and interferon-γ by CD4^+^ T cells in patients with inflammatory arthritis

**DOI:** 10.1111/cei.12434

**Published:** 2015-02-16

**Authors:** C Wu, J C Goodall, R Busch, J S H Gaston

**Affiliations:** 1 Department of Medicine, University of CambridgeCambridge, UK; 2 Department of Life Sciences, University of RoehamptonLondon, UK; 3 Department of Rheumatology and Immunology, The First Affiliated Hospital of China Medical UniversityShenyang, China

**Keywords:** immunophenotyping, inflammatory arthritis, MelCAM/MCAM/CD146, T helper cell subsets

## Abstract

Expression of the adhesion molecule, CD146/MCAM/MelCAM, on T cells has been associated with recent activation, memory subsets and T helper type 17 (Th17) effector function, and is elevated in inflammatory arthritis. Th17 cells have been implicated in the pathogenesis of rheumatoid arthritis (RA) and spondyloarthritides (SpA). Here, we compared the expression of CD146 on CD4^+^ T cells between healthy donors (HD) and patients with RA and SpA [ankylosing spondylitis (AS) or psoriatic arthritis (PsA)] and examined correlations with surface markers and cytokine secretion. Peripheral blood mononuclear cells (PBMC) were obtained from patients and controls, and synovial fluid mononuclear cells (SFMC) from patients. Cytokine production [elicited by phorbol myristate acetate (PMA)/ionomycin] and surface phenotypes were evaluated by flow cytometry. CD146^+^CD4^+^ and interleukin (IL)-17^+^CD4^+^ T cell frequencies were increased in PBMC of PsA patients, compared with HD, and in SFMC compared with PBMC. CD146^+^CD4^+^ T cells were enriched for secretion of IL-17 [alone or with IL-22 or interferon (IFN)-γ] and for some putative Th17-associated surface markers (CD161 and CCR6), but not others (CD26 and IL-23 receptor). CD4^+^ T cells producing IL-22 or IFN-γ without IL-17 were also present in the CD146^+^ subset, although their enrichment was less marked. Moreover, a majority of cells secreting these cytokines lacked CD146. Thus, CD146 is not a sensitive or specific marker of Th17 cells, but rather correlates with heterogeneous cytokine secretion by subsets of CD4^+^ helper T cells.

## Introduction

Interleukin (IL)-17-secreting T helper type 17 (Th17) cells produce a distinct set of proinflammatory cytokines, including IL-17A, IL-17F and IL-21, which normally regulate immunity at intestinal barriers and enable defence against extracellular bacteria and fungi [1]. Th17 cells differentiate from naive CD4^+^ T cells under the combined control of proinflammatory myeloid- and tissue-derived cytokines, such as IL-1, IL-6, transforming growth factor (TGF)-β and/or IL-23. They express characteristic surface markers, such as the chemokine receptors, CCR6 and CCR4, the receptor for IL-23 and CD161 [2,3]. The sensitivity and specificity of these and other markers for Th17 cells continue to be investigated.

Th17 cells have been implicated in a wide range of autoimmune and inflammatory diseases [4]. Evidence for their importance in pathogenesis comes from gene ablation and cell transfer studies in animal models, genomewide association studies and phenotypical and functional analysis of patients' T cells. However, Th17 responses appear to be dispensable for other autoimmune diseases, such as type 1 diabetes [5]. Among rheumatological conditions with a recognized Th17 component are rheumatoid arthritis (RA) and the spondyloarthritides (SpAs), a group of related conditions including ankylosing spondylitis (AS), psoriatic arthritis (PsA), arthritis related to inflammatory bowel disease, reactive arthritis and a subgroup of juvenile idiopathic arthritis.

In human inflammatory arthritides and in mouse models of these diseases, Th17 cells contribute to tissue inflammation; the IL-17 they produce plays an important role in recruitment of other leucocytes to sites of inflammation [6]. In patients with SpA, an increased frequency of Th17 cells is found in the peripheral circulation, with further enrichment in affected joints [7–10]. Genomewide association studies suggest altered regulation of Th17 differentiation or fate in AS [11]. Higher levels of IL-23 or increased IL-23R signalling could contribute [12]. Another potential mechanism involves signalling by misfolded B27 to Th17 cells, by aberrant interactions with surface receptors [13] or by induction of IL-23 secretion due to endoplasmic reticulum stress. Anti-IL-17 monoclonals have shown early signs of therapeutic efficacy in PsA and AS, indicating a key role for this cytokine [14]. Similarly, animal models, as well as phenotypical and functional studies and clinical trials in humans, point towards derangement of Th17 cells in RA [6,7,14]. Genomic evidence for specific Th17 involvement in RA is less extensive, but polymorphisms in the IL-6 signalling pathway and in the Th17-related chemokine receptor, CCR6, have been reported; other gene associations point towards suboptimal regulation of T cell activation [15].

More recent work has highlighted the heterogeneity of IL-17-secreting helper T cells. Many IL-17-producing cells are unable to produce interferon (IFN)-γ or IL-22, which used to be thought of as defining separate Th1 and Th22 effector cell populations, respectively. However, other IL-17-secreting CD4^+^ T cells do produce these cytokines, suggesting heterogeneity or plasticity of Th17 cell cytokine secretion patterns [7]. *In-vitro* studies and fate-mapping experiments in mice have demonstrated that Th17 cells can acquire IFN-γ secretion, and subsequently lose the ability to secrete IL-17 (‘ex-Th17 cells’), under the influence of IL-23 and chronic antigenic stimulation [16]. IL-17^+^ IFN-γ^−^ Th17 cells have been associated with anti-bacterial immunity, whereas autoimmunity may be associated with ‘bifunctional’, IL-17^+^ IFN-γ^+^ and/or IL-17^+^ IL-22^+^ CD4 T cells [17].

CD146/melanoma cell adhesion molecule (MelCAM) is an immunoglobulin (Ig) superfamily molecule, which is highly expressed at tight junctions of endothelial cells and on surfaces of vascular smooth muscle and trophoblast cells. CD146 has important functions in adhesion, tissue invasion and signalling [18,19]. In the human immune system, it is expressed on ≈2% of circulating T cells *ex vivo* [20] and induced upon polyclonal activation *in vitro* [21]. Circulating CD146^+^CD4^+^ T cells have an effector memory phenotype, expressing CD45RO but not CCR7; they are enriched for markers of recent activation *ex vivo* [22]. These studies, alongside endothelial adhesion experiments [22–24], suggest an important role for CD146 in transendothelial migration of certain activated Th cell subsets to sites of inflammation. A recent study showed that laminin-411 in vascular basement membranes interacts with CD146 on T cells to facilitate transmigration to inflammatory sites [25].

Patients with some inflammatory diseases (Behçet's disease, sarcoidosis and Crohn's disease) have significantly higher frequencies of CD146^+^ T cells in peripheral blood than healthy individuals [26]. Patients with RA and other inflammatory arthritides also have elevated CD146^+^ T cell frequencies in blood, and even higher frequencies at sites of inflammation [21,27]. Similarly, in our recent study of patients with several autoimmune connective tissue diseases, CD146 expression on circulating T cells was associated with effector memory phenotypes, and increased frequencies were observed in a subset of patients with marked T cell activation [28]. High levels of soluble CD146 have been detected in synovial fluid of patients with RA [29], although this may be shed by activated endothelia, as well as infiltrating CD146^+^ T cells.

Several recent studies link CD146 expression with Th17 effector function. In multiple sclerosis, the frequency of CD146 expression is higher in Th17 cells than in Th1 cells [24]. Conversely, cells producing IL-17, IL-22 and (to a lesser extent) IFN-γ are enriched within the CD146^+^ subset of CD4^+^ T cells [25]; moreover, some of the transcripts selectively enriched in CD146^+^ T cells are Th17-related [22,26,27]. Some of these studies proposed that CD146 is a Th17-associated cell surface marker, either alone [25] or in combination with other markers [30]; others, however, were careful to point out that not all IL-17-secreting Th cells express CD146 [26]. Here, we examined the relationship between CD146 expression, production of IL-17 with or without IL-22 and IFN-γ and the expression of putative Th17-associated surface markers by CD4^+^T cells in patients with SpA (PsA and AS) and RA and in blood from healthy donors.

## Materials and methods

### Patients

Peripheral blood was obtained from 16 patients with PsA [seven female, nine male; age = 44 ± 11 years; mean ± standard deviation (s.d.)], 10 patients with AS (three female, seven male; mean age, 45 ± 16 years), 14 patients with RA (nine female, five male; mean age, 56 ± 17 years) and 22 healthy donors (HDs; eight female, 14 male; mean age, 38 ± 10 years). Mean ages did not differ significantly between groups (*P* > 0·05), except for RA *versus* HD [*P* = 0·001, one-way analysis of variance (anova) with Holm–Sidak post-test for all pairwise comparisons]. Synovial fluid was collected, in most instances on separate occasions, from 10 patients with PsA, four with AS and four with RA. Patients met CASPAR (ClASsification criteria for Psoriatic Arthritis) classification criteria for PsA [31], the modified New York criteria for AS [32] or the 2010 American College of Rheumatology/European League Against Rheumatism classification criteria for RA [33]. Patients were recruited through out-patient clinics at Addenbrooke's Hospital, Cambridge, UK. They were being treated with biologicals, anti-inflammatory and immunosuppressive agents, and (in some RA patients) corticosteroids, alone or in combination. Erythrocyte sedimentation rate (ESR) and C-reactive protein (CRP) were measured at the time of venepuncture in a majority of patients. The study was approved by Cambridge University Hospitals' Regional Ethical Committee, and written informed consent was given by all patients.

### Preparation and stimulation of mononuclear cells

Peripheral blood and synovial fluid mononuclear cells (PBMC and SFMCs, respectively) were prepared by centrifugation over a Ficoll-Hypaque gradient (Amersham Pharmacia Biotech, Little Chalfont, UK) and cryopreserved in 10% dimethylsulphoxide (DMSO) in fetal bovine serum (FBS). Thawed cells were routinely >90% viable by Trypan Blue exclusion.

For cytokine secretion assays, PBMC were thawed in warm media and adjusted to a final concentration of 2 × 10^6^/ml in RPMI-1640 medium supplemented with 10% v/v heat-inactivated fetal calf serum, 2 mM glutamine, penicillin/streptomycin and 20 mM HEPES. The cells were seeded into 24-well culture plates (Nunc, Naperville, IL, USA), stimulated with phorbol myristate acetate (PMA; 50 ng/ml; Sigma, St Louis, MO, USA) and calcium ionomycin (1 μg/ml) (Sigma) for 5 h, and treated with GolgiStop monensin at 2·25 μM final concentration (Becton Dickinson, Mountain View, CA, USA), at the same time as the stimulation.

### Immunofluorescent staining and flow cytometry

Eight-colour flow cytometry was used to analyse the surface phenotype and intracellular cytokine production of PBMC and SFMC. The antibodies used were as follows: for surface staining, allophycocyanin-cyanin 7 (APC-Cy7)-labelled anti-CD3 (Becton Dickinson), biotin-labelled anti-CD4 (Biolegend, San Diego, CA, USA) used with Qdot655-streptavidin (Invitrogen, Carlsbad, CA, USA), phycoerythrin (PE)-labelled anti-CD146 (Becton Dickinson, Franklin Lakes, NJ, USA), fluorescein isothiocyanate (FITC)-labelled anti-CD161 (Biolegend), peridinin chlorophyll (PerCP)-Cy5·5-labelled anti-CCR6 (Becton Dickinson), FITC-labelled anti-CD26 (Becton Dickinson), PE-Cy5-labelled anti-CXCR3 (Becton Dickinson) and APC-labelled anti-IL-23R (R&D Systems, Minneapolis, MN, USA); for intracellular cytokine detection, PE-Cy7-labelled anti-IL-17A (Becton Dickinson), effluor660-labelled anti-IL-22 (eBioscience) and Pacific Blue-labelled anti-IFN-γ (eBioscience, San Diego, CA, USA). Appropriately conjugated IgG antibodies were used as isotype controls.

Cells were first stained with antibodies against surface antigens and then fixed and permeabilized using Perm/Fix solution (Becton Dickinson). Cells were washed with Perm/Wash buffer (Becton Dickinson) and stained with antibodies against intracellular cytokines. Flow cytometric analysis was performed using a FACSCanto II analyser (Becton Dickinson). CD3^+^CD4^+^T lymphocytes or their CD146^+^ and CD146^−^ subsets were gated within a scatter gate drawn narrowly on intact lymphocytes and frequencies of cells expressing surface markers or cytokines of interest, alone or in combination, were determined using quadrant statistics in FlowJo version 7·6 software (Tree Star, Inc., Ashland, OR, USA).

### Statistical analysis

Statistical analyses were performed using Prism (version 5·0; GraphPad Software, San Diego, CA, USA). Generally, medians and interquartile ranges were used as summary statistics, and non-parametric tests (Mann–Whitney *U*-test or Kruskal–Wallis anova with Dunn's post-test for multiple comparisons) were used for comparison between groups. To evaluate the relationship of CD146 to other markers in different patient groups, two-way anova was performed using log-transformed data, if necessary, to equalize variances; geometric mean frequencies were reported with 95% confidence intervals. Pearson's linear regression was used to correlate immunophenotypes with each other and with inflammatory markers.

## Results

### Circulating CD146^+^ CD4^+^ T cells are elevated in some patients with arthritis

**Fig. 1:**
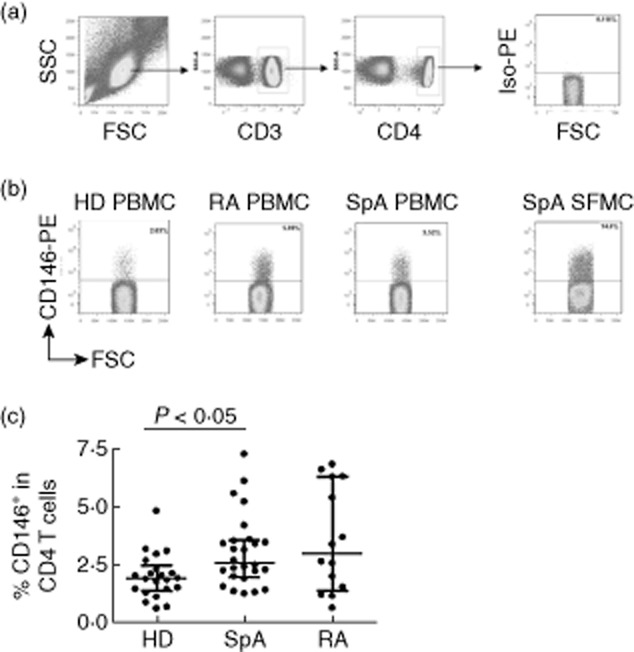
Enumeration of CD146^+^ CD4^+^ T cells *ex vivo* in patients and controls. (a) Gating of CD4^+^ CD3^+^ T lymphocytes, and isotype control for CD146 staining in this population. (b) Examples of CD146 staining on gated CD4^+^ T cells in representative patients and controls. (c) Frequencies of CD4 T cells expressing CD146, enumerated as in (b), in different study populations. Analysis by Kruskal–Wallis analysis of variance (anova) on ranks (*P* = 0·016). Individual data, means and interquartile ranges, and significant differences between groups (Dunn's post-test for multiple comparisons) are shown.

In order to quantify CD146 expression by CD4 T cells, PBMCs were isolated from patients with RA or SpA and from healthy controls, and cryopreserved. CD3^+^CD4^+^ cells were identified within a lymphocyte scatter gate (Fig. 1a) and the percentage of CD146^+^ cells determined within this population (Fig. 1b). CD146 staining appeared specific compared with isotype controls (Fig. 1a). This analysis was performed in PBMCs from HDs (*n* = 22), patients with SpA (AS, *n* = 10; or PsA, *n* = 16) and patients with RA (*n* = 14). Representative examples of CD146 staining are shown in Fig. 1b and a summary in Fig. 1c. Some patients with inflammatory arthritis had markedly higher frequencies of CD146^+^ T cells in the CD4 subset than healthy controls. The median increase above HDs was statistically significant in SpA patients, analysed together (*P* < 0·05, Kruskal–Wallis anova with Dunn's post-test; Fig. 1c). When treated as separate groups, the increase reached statistical significance in PsA (*P* < 0·05), but not in AS, the smallest of the patient groups (data not shown).

The same analysis showed no significant increase in median CD146 frequency in RA patients, but six of 14 RA patients had CD146^+^CD4^+^ T cell frequencies above 3·5%, whereas only one of 22 HDs exceeded this value (*P* = 0·0083, Fisher's exact test). (Note that the use of this cut-off is based on the distribution observed in HDs, which was consistent with previous studies from our laboratory [28].) In conclusion, increased frequencies of CD146^+^CD4^+^ cells are seen in a proportion of patients with inflammatory arthritis.

**Fig. 2:**
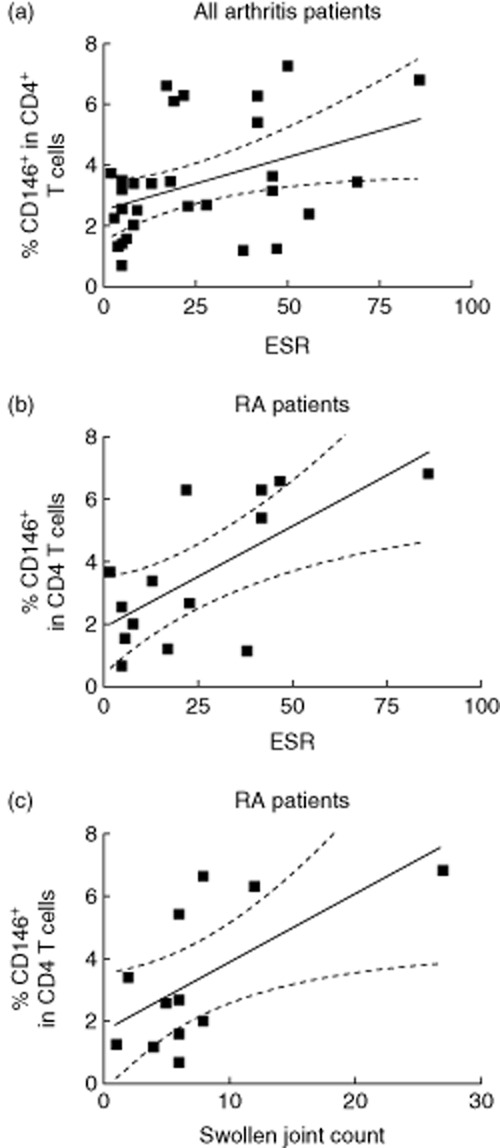
Clinical correlations of peripheral blood CD146^+^CD4^+^ T cell frequencies. (a) Correlation with erythrocyte sedimentation rate (ESR) for all patients with ankylosing spondylitis (AS), psoriatic arthritis (PsA) and rheumatoid arthritis (RA). Pearson's linear regression was used (best-fitting line shown with 95% confidence range; see text for *r*^2^ and *P*-value for non-zero slope). (b) Correlation with ESR for RA patients. (c) Correlation with swollen joint count for RA patients.

The small size of the study group limited clinical correlations, but the frequencies of CD146^+^CD4^+^ T cells correlated with erythrocyte sedimentation rate (ESR; *r*^2^ = 0·17, *P* = 0·022 for all patients with arthritis; Fig. 2a). There was no significant correlation with C-reactive protein (CRP), however (data not shown). When RA patients alone were analysed, the frequencies of CD146^+^CD4^+^ T cells correlated with ESR (*r*^2^ = 0·46, *P* = 0·008) and with swollen joint count (*r*^2^ = 0·41, *P* = 0·024; Fig. 2b,c). These data suggest that the CD146^+^ phenotype of circulating CD4^+^ T cells is related to the inflammatory process.

### IL-17, IL-22 and IFN-γ secretion is differentially enriched in the CD146^+^ Th subset

**Fig. 3:**
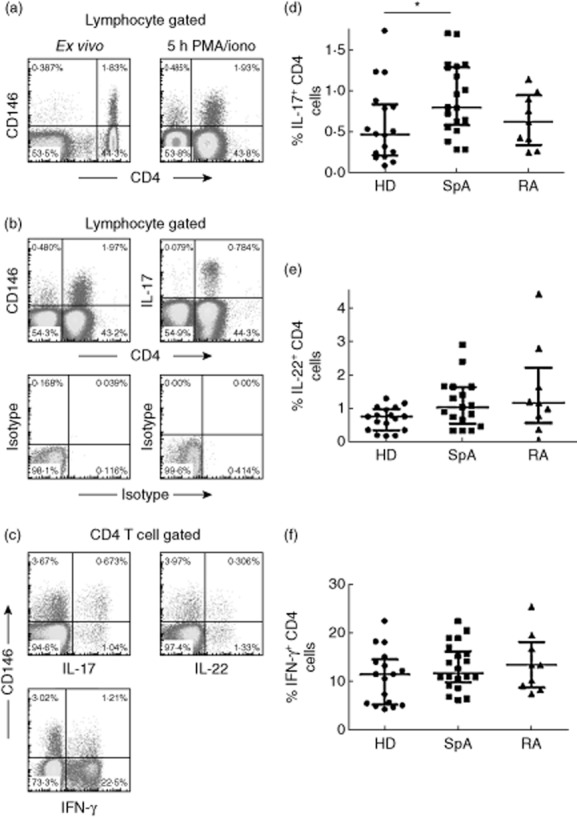
CD146 expression *versus* cytokine secretion by CD4^+^ T cells. (a) CD146^+^ T cell frequencies remain unaffected 5 h after stimulation with phorbol myristate acetate (PMA) and ionomycin in the presence of GolgiStop. Representative example of CD4 versus CD146 staining of peripheral blood lymphocytes from a spondyloarthritis (SpA) patient, stained either *ex vivo* or after *in-vitro* stimulation. Down-regulation of CD4 was observed post-stimulation, but did not affect the result. See Fig. S1 for statistics. (b) Representative example of co-staining for CD4 versus CD146 or interleukin (IL)-17 staining of peripheral blood lymphocytes from the same SpA patient after stimulation. The corresponding isotype controls are shown in the bottom row. (c) Co-staining of CD146 *versus* cytokines in stimulated CD4^+^ T cells from a representative SpA patient. The same SpA patient as in (a) and (b) was analysed, but with gating on CD3^+^CD4^+^ T cells, rather than on total lymphocytes, hence the higher frequencies of CD146 expression and IL-17 secretion. (d–f) Summary of frequencies of CD4^+^ T cells expressing IL-17 (d), IL-22 (e) or interferon (IFN)-γ (f) in patients and controls. Each panel shows individual data, medians and interquartile ranges, and was analysed by Kruskal–Wallis analysis of variance (anova) with Dunn's post-test (*P < 0·05).

In order to examine the relationship of CD146 expression with effector cytokine expression, PBMCs were stimulated with PMA/ionomycin in the presence of monensin for 5 h; surface-stained for CD3, CD4 and CD146; fixed and permeabilized; and stained for intracellular IL-17, IL-22 and IFN-γ. CD146 expression is known to be induced following *in-vitro* activation for >16 h [18,28], but no CD146 induction was detected at 5 h, when compared with *ex-vivo* surface-staining. A representative example from an SpA patient is shown in Fig. 3a. Supporting information, Fig. S1, shows a close correlation between CD146^+^ CD4 cell frequencies *ex vivo* and post-stimulation (Supporting information, Fig. S1a). No significant effect of stimulation was detected, whether in a pooled analysis of paired data (Supporting information, Fig. S1b) or after stratification into patient groups (Supporting information, Fig. S1c).

In all groups, a majority of CD146^+^ peripheral blood lymphocytes, and almost all IL-17-secreting lymphocytes, were within the CD4^+^ population; a representative example from the same SpA patient is shown in Fig. 3b. Secretion of IL-17, IL-22 and IFN-γ by CD4^+^ T cells was readily detectable (examples shown for the same patient in Fig. 3c). The median frequency of IL-17-secreting CD4^+^ T cells was approximately doubled in SpA compared with HDs (*P* < 0·05), but the difference in RA was not statistically significant (Fig. 3d). Within the SpA group, both PsA and AS patients showed increased frequencies of IL-17-secreting cells (data not shown). Increased secretion of IL-22 and IFN-γ by CD4^+^ T cells in arthritis patients was less pronounced and did not reach statistical significance (Fig. 3e,f).

**Fig. 4:**
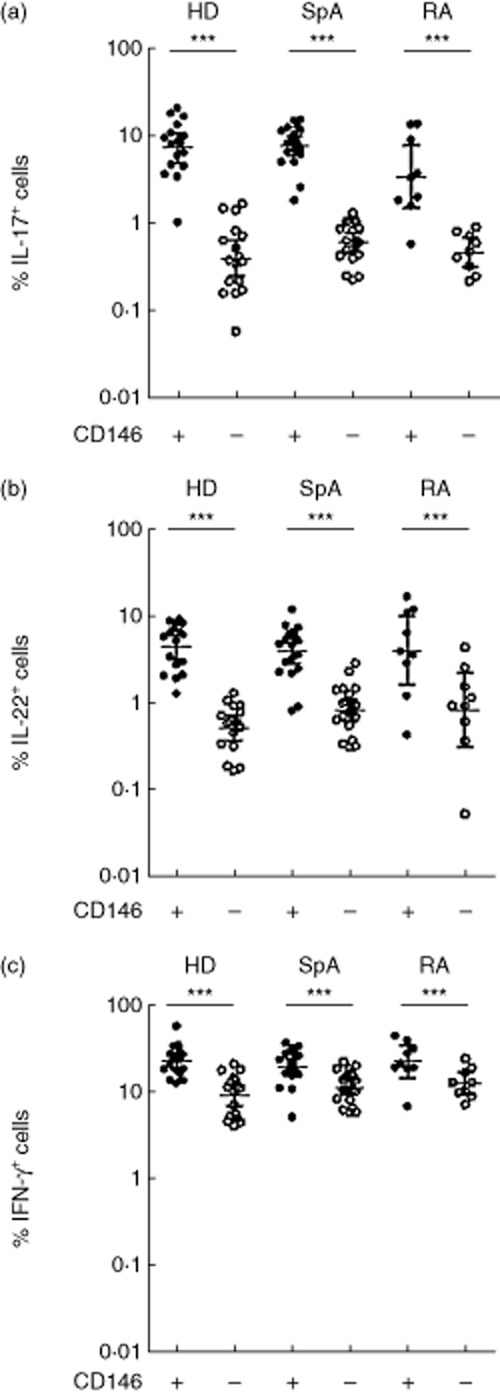
Mutual associations of CD146 expression and cytokine secretion by CD4 T cells. (a–c) Frequencies of cytokine-secreting CD4^+^ T cells [producing interleukin (IL)-17 (a), IL-22 (b) or interferon (IFN)-γ (c)] in the CD146^+^ *versus* CD146^−^ subset, analysed in patients and controls. Data were displayed on the same axis for ease of comparison between panels, log-transformed to equalize variances, and analysed using two-way analysis of variance (anova); CD146^+^ and CD146^−^ data from each patient were matched. Individual data, geometric means and 95% confidence intervals are shown. Asterisks indicate significant enrichment in the CD146^+^ subset using Dunn's post-test for multiple comparisons (**P < 0·01; ***P < 0·001). Disease effects were not significant.

Flow cytometry clearly showed that effector cytokines were secreted both by CD146^+^ and CD146^−^CD4^+^ T cells (Fig. 3c). If CD146 and cytokines are expressed independently of each other, then the percentage of cytokine-expressing CD4 T cells should not differ between CD146^+^ and CD146^−^ cells. However, a much greater proportion of CD146^+^ than CD146^−^CD4 T cells produced IL-17, both in HDs and in patients with SpA and RA (Fig. 4a). The enrichment of IL-17 secretors in the CD146^+^ population was ≈18-fold in HDs, ≈13-fold in SpA and ≈7·4-fold in RA (geometric means). The overall increase in Th17 frequencies in SpA was not related specifically to either the CD146^+^ or the CD146^−^ subset.

Similar analyses were performed for other effector cytokines. IL-22 secretion by CD4 T cells was associated significantly with CD146 expression, although the fold enrichments were less (8·5, 4·8 and 4·8, respectively, in the HD, SpA and RA groups; Fig. 4b). Even less enrichment within the CD146^+^ CD4 subset was observed for IFN-γ (2·5-, 1·7- and 1·8-fold in the three groups; Fig. 4c). However, the frequency of IFN-γ producers was much higher overall; therefore, in the CD146^+^ subset, IFN-γ producers were at least as abundant as IL-17 producers.

Conversely, we asked what proportion of cytokine-producing cells expressed CD146. CD146^+^CD4^+^ T cells accounted, on average, for ≈18%–31% of IL-17-producing Th cells, both in arthritis patients and HDs (Table 1). Similarly, CD146^+^ cells comprised just greater than 10% of IL-22-producing CD4^+^ T cells, and approximately 3–5% of IFN-γ producers, in patients and controls (data not shown). Thus, CD146 was not a sensitive cell surface marker of Th effector cells in general, nor of any one Th subset in particular.

**Table 1:** Co-expression of CD146 with either CCR6 or CD161 in peripheral blood: comparison between interleukin (IL)-17^+^ and IL-17^−^ T helper cells

		% in IL-17^+^CD4^+^ T cells		% in IL-17^–^CD4^+^ T cells		Ratio	
Group	Marker (combination)	Median	IQR	Median	IQR	Median	IQR
HD (*n* = 17)	CD146^+^	18·1	(14·1–28·3)	1·7	(1·4–2·5)	10·9	(9–12·1)
	CCR6^+^	83·1	(66·4–90·7)	18·4	(14·1–33·5)	2·9	(2·0–5·9)
	CD146^+^CCR6^+^	16·5	(13·4–26·5)	1·1	(0·6–1·3)	20·8	(11·5–24·2)
	CD161^+^	53·8	(39·1–60·6)	13·9	(10·6–19·3)	3·3	(2·7–4·5)
	CD146^+^CD161^+^	13·4	(10·6–17·4)	0·8	(0·5–1·1)	15·3	(11·8–21·3)
PsA (*n* = 12)	CD146^+^	31·0	(23·8–38·7)	2·7	(2·0–4·0)	9·9	(9·3–10·9)
	CCR6^+^	73·8	(63·0–80·7)	30·0	(22·7–32·7)	2·5	(2·1–2·9)
	CD146^+^CCR6^+^	21·7	(17·5–28·3)	1·7	(1·1–2·5)	14·1	(10·2–16·2)
	CD161^+^	52·8	(41·3–63·7)	13·3	(9·3–19·8)	3·8	(3·2–5·1)
	CD146^+^CD161^+^	18·2	(14·6–22·4)	1·0	(0·5–1·7)	15·0	(11·4–20·3)
AS (*n* = 7)	CD146^+^	31·1	(26·0–35·8)	3·2	(3·0–4·0)	8·7	(6·8–10·2)
	CCR6^+^	88·1	(85·9–94·1)	20·6	(17·3–31·5)	4·3	(3·0–4·9)
	CD146^+^CCR6^+^	27·6	(26·7–33·0)	1·3	(1·2–2·8)	21·2	(12·4–24·3)
	CD161^+^	45·5	(39·5–58·2)	12·1	(11·3–15·5)	3·3	(2·6–4·0)
	CD146^+^CD161^+^	14·1	(9·7–17·7)	1·0	(0·7–1·3)	9·1	(7·1–19·5)
RA (*n* = 8)	CD146^+^	30·9	(23·6–34·5)	3·0	(2·6–4·4)	8·7	(7·3–11·2)
	CCR6^+^	74·5	(65·4–82·4)	20·6	(15·8–35·6)	3·8	(2·3–5·2)
	CD146^+^CCR6^+^	24·4	(20·5–29·5)	1·3	(1·0–2·3)	18·3	(7·5–26·9)
	CD161^+^	55·2	(37·0–63·8)	11·7	(10·6–14·4)	4·3	(3·7–5·3)
	CD146^+^CD161^+^	17·9	(13·4–20·1)	1·0	(0·8–1·2)	17·7	(14·7–21·5)

**Fig. 5:**
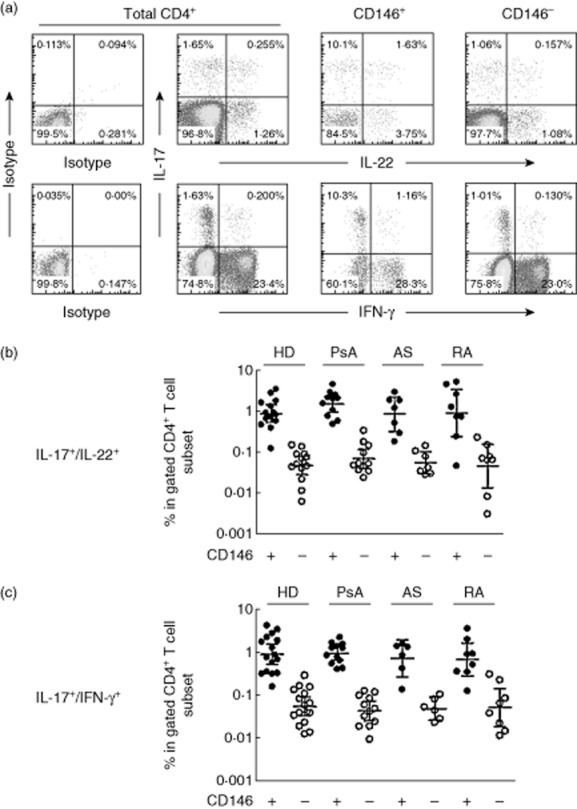
CD4^+^ CD146^+^ T cells are enriched for dual cytokine-secreting cells. (a) Representative analysis of CD4^+^ T cells from a spondyloarthritis (SpA) patient, showing isotype control staining or co-staining for interleukin (IL)-17 with either IL-22 or interferon (IFN)-γ. The analyses were gated either on total CD4^+^ T cells (left) or on the CD146^+^ and CD146^−^ subsets within this population (right), as indicated. (b) Frequencies of IL-17^+^IL-22^+^ cells, compared in each group of patients or healthy donors (HDs) between CD146^+^ and CD146^−^ CD4 T cells. Individual data, geometric means and 95% confidence intervals are shown on a log scale. Data were log-transformed to equalize variances and analysed using two-way analysis of variance (anova), with matching of CD146^+^ and CD146^−^ data from each patient. The effect of CD146 was significant in each patient group (*P* < 0·001, Dunn's post-test). There were no significant differences between patient groups. (c) Frequencies of IL-17^+^ IFN-γ^+^ cells, analysed as in (b). The enrichment of dual cytokine secretors in the CD146^+^ population was significant (*P* < 0·001) in each patient group. There were no significant differences between patient groups.

Peripheral blood mononuclear cells (PBMCs) from patients or controls were analysed. CD4^+^IL-17^+^ (left column) or CD4^+^IL-17^−^ T cells (middle column) were gated and percentages of cells with the indicated phenotypes enumerated. Values for CD146^+^ cells represent the average of several measurements, each in combination with a different surface marker. The ratio of percentages (IL-17^+^/IL-17^−^) is shown in the right-most column. Medians are shown with interquartile ranges (IQR) in parentheses. The slight discrepancies between the median percentages of IL-17^+^ T helper (Th) cells expressing CCR6 and CD161 reported in this Table and in Fig. 5 arose because of different sample sizes (not all patients were analysed for co-expression), and because slightly different cut-offs for positivity were used. HD = healthy donors; PsA = psoriatic arthritis; AS = ankylosing spondylitis; RA = rheumatoid arthritis.

### Dual cytokine secretion in the CD146^+^ subset of circulating CD4^+^ T cells

Individual human effector Th cells may secrete multiple cytokines, including combinations that, historically, had been classified as belonging to distinct helper cell subsets (e.g. IL-17 with the Th1 ‘signature cytokine’, IFN-γ). In our study, approximately 15–20% of IL-17-secreting CD4 T cells also secreted IL-22 or IFN-γ, as shown for a representative SpA patient in Fig. 5a (left panels). Accordingly, in a proportion of patients and controls, we compared the frequencies of dual cytokine-secreting cells (upper right quadrants) between CD146^+^ and CD146^−^CD4 T cells (Fig. 5a, right and far right panels). In HDs and the different patient groups, IL-17/IL-22 dual secretors were enriched by 15–22-fold within the CD146^+^ subset (geometric means), compared to CD146^−^ cells (summarized in Fig. 5b). A similar degree of enrichment was seen for IL-17/IFN-γ dual secretors in the CD146^+^ subset (13–21-fold; Fig. 5c). These fold enrichments for bifunctionality were at least as high as for IL-17 producers overall (cf. Fig. 4a). Together, the data in Figs 4 and 5 suggest that effector cytokine secretion by CD146^+^CD4^+^ T cells is heterogeneous, and that CD146 is not a specific marker of Th-17-only cells, producing IL-17 without IFN-γ or IL-22.

### CD146^+^CD4^+^ circulating T cells are enriched in some putative Th17-related cell surface markers

The expression of selected putative Th17 surface markers (CCR6, CD26, CD161 and IL-23R, with CXCR3 as a negative control) was compared between CD146^+^ and CD146^−^ CD4^+^ T cells, as well as with IL-17^+^CD4^+^ T cells.

**Fig. 6:**
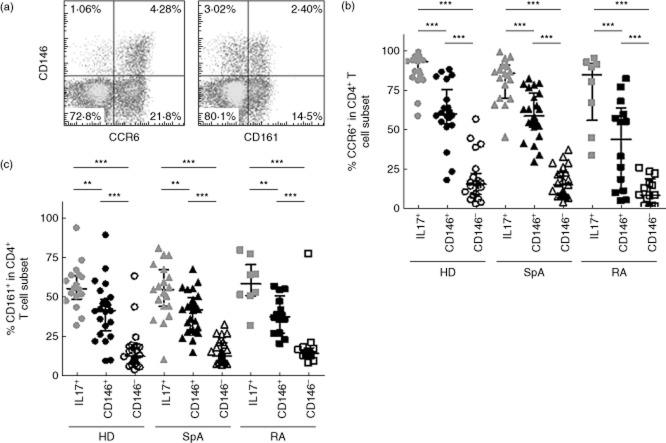
Association of CD146 expression with CCR6 and CD161. (a) Flow cytometry analysis of a representative spondyloarthritis (SpA) patient for CD146 *versus* CCR6 or CD161, gated on CD4^+^ T cells. (b) Frequency of CCR6-expressing cells in the interleukin (IL)-17-secreting CD4 cells (grey symbols), compared to CD146^+^ CD4 cells (black symbols) and CD146^−^ CD4 cells (open symbols) in healthy donors (HDs) (circles) and patients with SpA (triangles) and rheumatoid arthritis (RA) (squares). Individual data, medians and interquartile ranges are shown. Using two-way analysis of variance (anova), both the differences between CD4 cell subsets (*P* < 0·0001) and those between subject groups (*P* = 0·0004) were significant. Asterisks indicate significant pairwise differences between subsets (***P < 0·001; Dunn's post-test). (c) Frequencies of CD161^+^ cells, analysed as in (b). Using two-way anova, the differences between CD4 cell subsets (*P* < 0·0001) were significant, but there was no significant disease effect (*P* > 0·05). Asterisks indicate significant pairwise differences between subsets (**P < 0·01; ***P < 0·001; Dunn's post-test).

CD146 was co-expressed with CCR6 and CD161, as shown for a representative SpA patient in Fig. 6a. Compared to CD146^−^ cells, CD146^+^ cells were significantly enriched for CCR6 and CD161: 40–60% of CD146^+^ cells were CCR6^+^ and 30–40% were CD161^+^ (Fig. 6b,c). CCR6 (but not CD161) expression also differed between patient groups (lower expression in the arthritides; *P* = 0·0004), although this effect was smaller in magnitude (Fig. 6b).

An even greater majority of IL-17-secreting CD4^+^ T cells expressed CCR6; approximately half also expressed CD161 (Fig. 6b,c). Both CCR6 and CD161 were thus expressed by a greater proportion of IL-17^+^CD4^+^ T cells than CD146 (18–31%; Table 1). Thus, of these markers, CCR6 was the most sensitive individual marker of IL-17 production by CD4^+^ T cells, followed by CD161 and CD146.

However, CD161 and CCR6 exhibited lower specificity for Th17 cells than CD146: a significant proportion of IL-17-negative cells expressed these markers, and their enrichment within the IL-17-secreting cells was only 3–4-fold, much lower than the ≈9–11-fold enrichment observed for CD146 (Table 1).

Other putative Th17 markers were not associated with CD146 expression (Supporting information, Fig. S2). CD26 was present on ≈70% of both CD146^+^ and CD146^−^ cells, and even more highly expressed (≈80%) on IL-17-secreting cells (Supporting information, Fig. S2a). CXCR3, which is not related to Th17 cells, was expressed on a minority of CD4 T cells, regardless of CD146 expression (Supporting information, Fig. S2b). (The relationship of CXCR3 expression to IL-17 could not be evaluated, because stimulation with PMA and ionomycin resulted in rapid down-regulation of CXCR3 expression on all CD4^+^ cells.) The receptor for IL-23, a key cytokine for differentiation, expansion and maintenance of Th17 cells, was expressed on only a minority of IL-17-producing CD4 cells, and in similar, low proportions of CD4 cells with or without CD146 (Supporting information, Fig. S2c). In conclusion, only some surface markers previously implicated in Th17 differentiation or function were expressed selectively on the CD146-expressing subset.

**Fig. 7:**
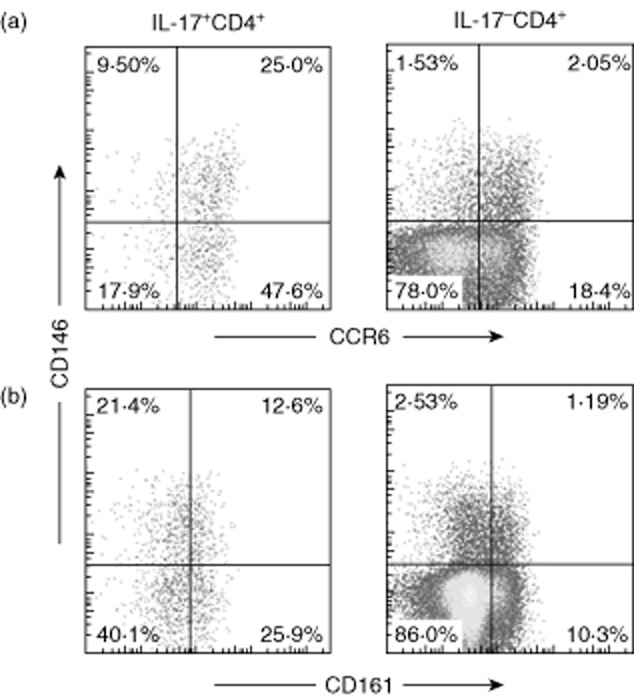
Relationship of effector cytokine secretion to coexpression of CD146 with CCR6 or CD161. Analysis of a representative spondyloarthritis (SpA) patient's peripheral blood. Co-expression of CD146 with CCR6 (a) or with CD161 (b) is shown, gated either on IL-17-producing (left panels) or non-producing (right panels) CD4^+^ T cells. Descriptive statistics for the different patient groups are summarized in Table 1.

In addition, we investigated the relationship between IL-17 secretion and CD146 co-expression with either CCR6 or CD161. IL-17^+^CD4^+^ or IL-17^−^CD4^+^ T cells were gated, and expression of these surface markers was evaluated (Table 1, with representative examples in Fig. 7). In healthy donors, CD146^+^CCR6^+^ cells were enriched approximately 21-fold among IL-17-producing (compared to non-producing) Th cells (Table 1) – a much greater enrichment than observed for CD146 alone (11-fold) or CCR6 alone (3-fold). Similar trends were observed in arthritis patients (Table 1). Thus, co-expression of CCR6 with CD146 was more specific for IL-17-secreting Th cells than either of these markers individually. Inevitably, however, co-expression of CCR6 with CD146 was found on a slightly smaller percentage of IL-17-secreting cells than expression of CCR6 or CD146 individually (Table 1). Thus, the CCR6^+^CD146^+^ phenotype was less sensitive at identifying IL-17-secreting cells than the expression of either one of these markers. Similarly, co-expression of CD161 with CD146 was more specific and less sensitive for IL-17-secreting cells than either marker individually in patients and controls (Table 1). Given that CD26 and IL-23R individually were not associated with IL-17 secretion, it is not surprising that their co-expression with CD146 did not improve sensitivity or specificity in relation to IL-17 production (data not shown).

### Relationship between CD146 expression and cytokine secretion in synovial fluid

**Fig. 8:**
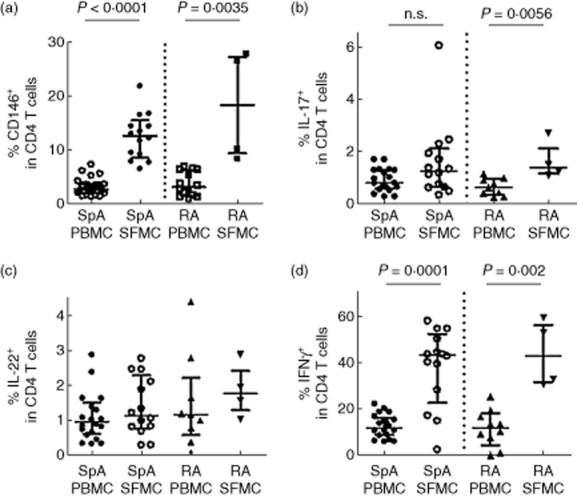
Frequencies of CD146^+^ and of cytokine-producing Th cells in synovial fluid. (a) CD146^+^ CD4 T-cell frequencies in peripheral blood (as in Fig. 1c) versus synovial fluid mononuclear cells from patients with spondyloarthritis (SpA) [ankylosing spondylitis (AS), *n* = 4; psoriatic arthritis (PsA), *n* = 10] and rheumatoid arthritis (RA) (*n* = 4). Individual data, medians and interquartile ranges are shown. Statistical significance of the enrichment in synovial fluid mononuclear cells (SFMC) was assessed for each group by Mann–Whitney *U*-test. (b–d) Comparison between peripheral blood and synovial fluid of frequencies of CD4^+^ T cells producing interleukin (IL)-17 (b), IL-22 (c) and interferon (IFN)-γ (d). Analysis as in (a).

Compared with peripheral blood, the frequency of CD146^+^CD4^+^ T cells was increased further in synovial fluid from joints of patients with SpA or RA (Fig. 8a). In the SpA group, results for PsA and AS were similar (data not shown). This was consistent with selective recruitment of CD146^+^CD4^+^ T cells to sites of inflammation. Cytokine-secreting cells were attracted to the synovial fluid compartment, albeit in a distinct pattern from that found in blood: there was only slight enrichment of IL-17-secreting cells in synovial fluid, no discernible enrichment for IL-22 and marked enrichment for IFN-γ production (Fig. 8b–d).

**Fig. 9:**
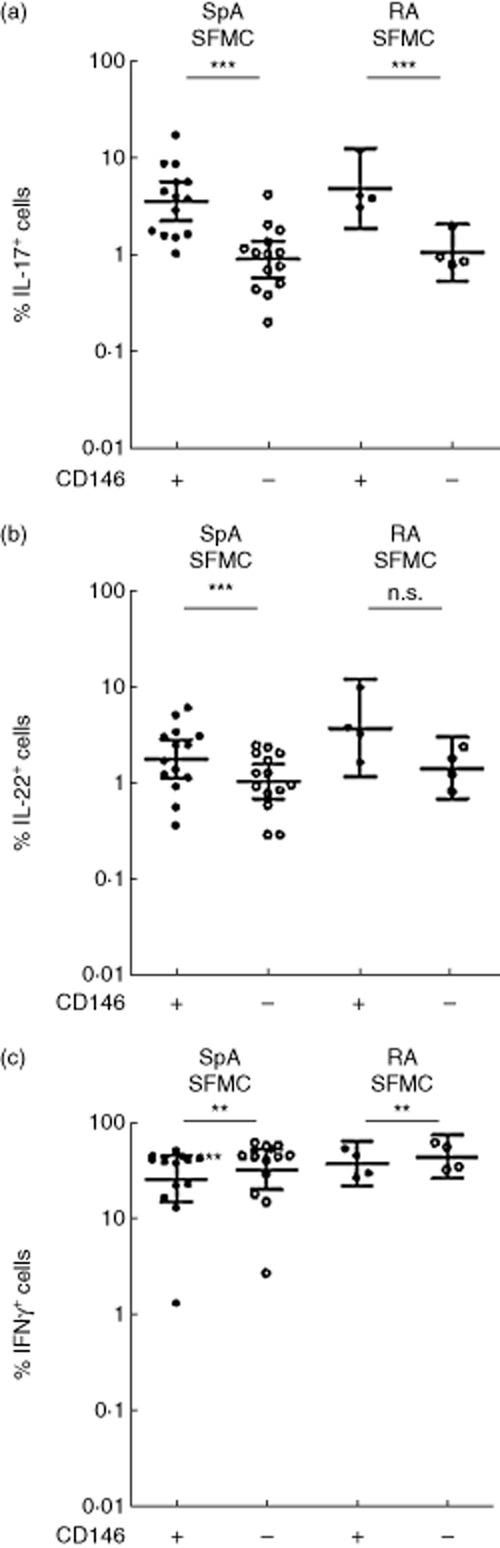
Cytokine production in CD146^+^ *versus* CD146^−^ synovial fluid T helper (Th) cells. (a)–(c) The percentages of cells expressing interleukin (IL)-17 (a), IL-22 (b), or interferon (IFN)-γ (c) were determined after gating on CD4^+^ cells with or without CD146, in synovial fluid mononuclear cells (SFMC) samples from patients with rheumatoid arthritis (RA) or spondyloarthritis (SpA). Individual values are shown with geometric means and 95% confidence intervals. For each patient group, data were log-transformed to equalize variances; differences between matched CD146^+^ and negative cells analysed by paired *t*-test (**P < 0·01; ***P < 0·0001).

If effector cytokine-secreting cells are recruited primarily to synovial fluid by CD146-dependent pathways, one might expect to find a greater enrichment of cytokine secretion within the CD146^+^ subset in synovial fluid than in peripheral blood. The opposite was observed (Fig. 9). In synovial fluid, IL-17-producing CD4 T cells were enriched within the CD146^+^ subset, but only by 4-fold in SpA patients and by approximately 4·6-fold in RA patients (geometric means; *versus* 13-fold and 7·4-fold in blood, respectively, see above). Comparing Fig. 4a with Fig. 9a, the main difference between peripheral blood and synovial fluid was that a greater proportion of CD146^−^ CD4^+^ cells secreted IL-17 in synovial fluid. IL-22 in synovial fluid also showed smaller enrichments within CD146^+^CD4 T cells (1·7- and 2·6-fold, respectively, in SpA and RA, Fig. 9b) than in peripheral blood (4·8-fold for both); for IFN-γ, the trend was even reversed (0·8- and 0·85-fold; Fig. 9c). Thus, even though CD146 may play a role in the recruitment of effector Th cells to synovial fluid, other factors must be at play to account for the fact that enrichment of cytokine-secreting effector cells in the CD146^+^ subset was less pronounced than in peripheral blood.

## Discussion

In this study, in patients with RA and two SpAs (AS and PsA), as well as in healthy controls, we investigated the relationship between IL-17 production (following brief polyclonal stimulation) and CD146 expression by CD4 T cells *ex vivo*. Both in patients and in healthy donors, CD146^+^ cells were enriched for IL-17-producing cells and for surface markers of Th17 cells. CD146^+^ cells were enriched even further in synovial fluid of SpA patients. The frequency of circulating CD146^+^CD4^+^ T cells was elevated above normal in some patients with arthritis, correlating with ESR. Together, these data are consistent with a contribution of CD146^+^ T cells to pathogenesis in inflammatory arthritis, similar to conclusions from previous studies in other autoimmune diseases (see Introduction). Our other findings, however, indicate that the relationship between Th17 effector function, CD146 expression and inflammatory recruitment is more complex than had been supposed previously. Importantly, CD146 by itself is neither a sensitive nor a specific marker of Th17 cells, and combinations with other markers, at best, improve specificity but reduce sensitivity. CD146^+^CD4^+^ T cells in blood and synovial fluid are heterogeneous in their patterns of IL-17, IFN-γ and IL-22 secretion; and CD146-dependent recruitment to sites of inflammation does not account for cytokine secretion patterns in synovial fluid.

Our results confirmed earlier findings [7–10] that Th17 cells are expanded in the blood of SpA patients. A similar trend in RA had been reported previously, but did not reach significance in our small RA cohort; this was also true for the previously observed increase in IL-22-producing CD4 cells in SpA. Both the present study and previous work showed that circulating Th1 cells are not expanded in SpA or RA. Together, these findings are consistent with the hypothesis that these inflammatory arthritides are driven in part by excessive Th17 activity.

Furthermore, we confirmed recent findings [27] that a subset of RA patients has increased frequencies of circulating CD146^+^CD4 T cells, and report significantly increased frequencies of CD146^+^ T cells in SpA. This phenotype correlated with ESR, albeit weakly. The RA group was older, on average, than healthy controls (see Materials and methods), but the ages of the other groups were not significantly different, and no systematic age effect on CD146^+^CD4^+^ T cell frequencies was noted (*P* > 0·05 for non-zero slope in each group by linear regression). Moreover, there was no marked effect of immunosuppressive therapies on CD146^+^CD4^+^ T cell frequencies or PBMC yields in patients. However, our study was not powered for a multivariate analysis, which would be required to address the effects of clinical and demographic variables more definitively. Given that the frequency of CD146^+^CD4^+^ T cells is elevated in various other autoimmune conditions, which are treated with different drug regimens, it seems likely that the increased frequencies seen in AS on average, and in some RA patients, reflect the autoimmune process, rather than the effect of immunosuppression.

Both in the blood and synovial fluid of arthritis patients and in the blood of HDs, circulating CD146^+^CD4^+^ T cells were enriched for cytokine-producing Th effector cells. This was expected from previous work showing relationships of CD146 expression to recent or chronic T helper cell activation and effector function (cf. Introduction). Importantly, the brief polyclonal stimulation protocol used here to elicit effector cytokine production did not perturb CD146 expression. Of the cytokines examined, the fold enrichment was greatest for IL-17, followed by IL-22, and least for IFN-γ; in synovial fluid, however, the corresponding hierarchy was different: IFN-γ > IL-17 > IL-22.

As we have shown previously in healthy donors and connective tissue disease patients [28], virtually all CD146^+^CD4 T cells are CD45RO^+^ memory cells, whereas CD146-negative CD4 cells include both memory and naive populations in proportions that vary between individuals but average approximately 50%. The admixture of naive CD4 T cells would account for the ≈2-fold enrichment for IFN-γ in the CD146^+^ population. In contrast, the much greater enrichments observed in blood for IL-17 and IL-22 must be due to coordinate expression of CD146 with these effector cytokines. Collectively, these findings are consistent with earlier reports that CD146^+^CD4 T cells are enriched for Th17 effector function and related transcripts [22,24,26,27]. Unlike blood, synovial fluid Th cells are predominantly memory cells, so admixture of naive cells causes little or no bias in this compartment [34].

As expected from earlier studies of patients with arthritis conditions (cf. Introduction), CD4^+^ T cells in arthritis patients' synovial fluid were enriched for CD146 expression compared to peripheral blood, supporting a role for CD146 in T cell recruitment to arthritic joints and other inflammatory sites. However, even in synovial fluid, CD146^+^ cells comprised only a minority of infiltrating CD4 cells. Moreover, IL-17- and IL-22-secreting cells were enriched little, if at all, and the association with CD146 was less pronounced in synovial fluid. CD146 could be down-regulated, shed or proteolysed following recruitment of CD4^+^CD146^+^ cytokine-secreting T cells to inflammatory sites; alternatively, CD146^−^ effector Th cells might be recruited by other extravasation mechanisms. Other compartments, such as synovial tissue, were not sampled in this study. In any case, the elevated blood frequencies of CD146^+^CD4^+^ T cells and IL-17-secreting Th cells in patients must reflect a net balance of entry into blood (following clonal expansion of activated cells in lymph nodes and/or at sites of inflammation) and recruitment to sites of inflammation.

Interfering with trafficking of pathogenic effector T cells to sites of inflammation is a potentially appealing therapeutic strategy. For example, some of the effects of TNF blockade have been reported to reflect decreased recruitment of cells to the joint, rather than simply neutralization of this proinflammatory cytokine in the joint [35]. Our observational studies, however, cast doubt on the therapeutic potential of CD146 blockade in SpA or RA, as this intervention may prevent only a minority of effector T cells from reaching inflamed joints. Although we did not perform functional adhesion assays in this study this conclusion is consistent with previous work in multiple sclerosis, where Th17 cells showed a similar degree of enrichment among CD146^+^CD4 cells, yet CD146 blockade diminished T cell/endothelial interactions by only a small (albeit statistically significant) amount [24].

Consistent with these findings, some putative Th17-associated surface markers were also enriched substantially in CD146^+^ CD4^+^ T cells. As reported previously [10,36], ≥80% of IL-17^+^CD4^+^T cells expressed CCR6, while ∼50% expressed CD161 [37] – more than the 20–25% of IL-17^+^CD4^+^ T cells which expressed CD146. Moreover, we found CCR6 and CD161 to be enriched in CD146^+^ cells, albeit less than in functional Th17 cells. CCR6 expression on CD146^+^ CD4 cells may contribute to their selective recruitment to synovial fluid, which is a source of the CCR6 ligand, CCL20 [38]. Several other putative Th17-related markers, however, were not enriched selectively in either IL-17-producing or CD146-expressing CD4 cells. Both subsets expressed CD26 at a high frequency, but so did most CD4 T cells lacking CD146. Perhaps more surprisingly, we found that only a minority of both subsets expressed IL-23R, a receptor that enables IL-23 signalling during Th17 differentiation and expansion. IL-23R may be lost in cells which initially differentiated in response to IL-23 and IL-6 (i.e. in the absence of TGFβ [39]), unless expression is stabilized by further exposure to TGF-β and IL-6 [1,2,40]. In any case, the close similarity in surface phenotypes between CD146^+^ and IL-17-producing CD4 T cells does not prove a functional relationship between these two phenotypes.

Even though the enrichment for IL-17 producers in the CD146^+^ subset was marked and robust in peripheral blood, our data showed that CD146 expression on T cells is neither a sensitive nor a specific marker of Th17 cells. Conceivably, the frequency of both phenotypes might have been underestimated for technical reasons, but stimulation and detection conditions were carefully optimized and results in healthy donors were consistent with earlier work. None the less, IL-17 was produced by only a minority of CD146^+^ cells under our stimulation conditions and, conversely, a majority of IL-17 producers lacked detectable CD146 expression. The combination of CD146 with either CCR6 or CD161 improved the specificity of association with IL-17 secretion, consistent with previous work [30], but reduced sensitivity. Two other putative Th17-related markers, CD26 and IL-23R, showed no association with either IL-17 secretion or CD146 expression, and co-expression of CD146 with these markers did not improve delineation of IL-17-secreting cells (not shown).

Moreover, T cells expressing other cytokines (IL-22 and IFN-γ), mainly without IL-17, were also enriched in the CD146^+^ subset. IFN-γ-secreting cells outnumbered IL-17-secreting cells in this population, and IL-22-secreting cells were only slightly less frequent. Thus, based on several lines of evidence, CD146 marked subsets of effector T helper cells with heterogeneous cytokine secretion patterns, with IL-17-secreting cells comprising a minority, albeit a substantial one.

Further complexity arises from the fact that effector Th cells may secrete combinations of cytokines and exhibit functional plasticity. In mice, fate-mapping studies have shown that repeated stimulation may result in activation of IFN-γ production in cells that previously produced only IL-17, followed by loss of IL-17 secretion and conversion to ‘ex-Th17’ cells [16,41]. In our study, a significant minority of IL-17-producing CD4 cells (approximately 15%) also secreted IFN-γ. These bifunctional Th1/17 cells were enriched to a similar degree within the CD146^+^ population as IL-17-secreting cells overall, whereas cells producing IFN-γ without IL-17 were much less highly enriched. These findings are consistent with the notion that the bifunctional cells were derived from Th17 cells; the IFN-γ-secreting cells may represent a mixture of ex-Th17 cells and conventional Th1 cells in unknown proportions. Similar considerations apply to cells secreting IL-22 with or without IL-17 [17]. These ideas will remain difficult to test directly, because fate-mapping and gene knock-out approaches are unavailable in humans, and gene expression signatures or marker profiles that distinguish conventionally differentiated Th17 cells [42] from bifunctional Th1/17 or Th17/22 cells, ex-Th17 cells and conventional Th1 and Th22 cells are only emerging in humans.

Functional heterogeneity explains why there was no significant correlation between CD146^+^ CD4 cell frequencies and Th17 frequencies in either HDs or in RA patients' blood (*P* > 0·05 each; Supporting information, Fig. S3a,b) – the presence of other effector cell populations within the CD146^+^ population and the lack of CD146 expression on many Th17 cells would confound any such correlation. Surprisingly, a statistically significant correlation was observed in SpA (Supporting information, Fig. S3c) and, within this group, appeared to be selective for PsA (data not shown). PsA patients showed the greatest increase both in Th17 and CD146^+^CD4 cell frequencies above healthy controls, so in these circumstances a correlation may emerge. Studies of larger patient populations would be required to confirm this finding and to assess whether this reflects disease-specific co-expression patterns, differences in the amount or quality of systemic inflammation or confounding effects of therapy. In any case, taken together these analyses confirmed that CD146 should not be considered a surrogate marker of Th17 effector function, even though Th17 cells are enriched substantially within this population. The correlation of CD146^+^CD4 T cell frequencies with inflammatory markers is also weak. Again, the likeliest explanation is the mixture of effector phenotypes present in this population, not all of which may be disease-relevant, and the possibility that effector Th cell recruitment to inflammatory sites is partially redundant.

To conclude, this study showed that the frequency of CD146^+^CD4^+^ T cells is increased in peripheral blood from patients with SpA and some patients with RA. In arthritis patients and controls, IL-17 production and Th17-associated surface markers are greatly enriched in CD146^+^CD4 cells, but the functional repertoire of CD146^+^CD4 cell populations includes secretion of IL-22 and IFN-γ, alone or with IL-17. CD146 may facilitate migration of Th cells, secreting various effector cytokines, from blood to the synovium through binding to endothelia, but many effector Th cells in inflamed joints lack CD146 and alternative recruitment mechanisms may exist. CD146 is neither a sensitive nor a specific marker of Th17 cells and, at best, a poor correlate of inflammation or disease activity in inflammatory arthritis. The relationships between CD146 expression and Th17 cytokine secretion may vary between patient populations, but further studies would be required to confirm this finding and assess its clinical significance.

## Supporting Information

**Fig. S1.** CD146 expression on CD4^+^ T cells is minimally perturbed following 5 hours of stimulation with PMA and ionomycin. **Fig. S2.** Relationship of CD146 expression with additional surface markers. **Fig. S3.** Correlations of IL-17 secretion with CD146 expression.
