# Supplementary figures and images for: Relationship of CD146 expression to secretion of interleukin (IL)-17, IL-22 and interferon-γ by CD4+ T cells in patients with inflammatory arthritis
Source: Clin Exp Immunol. 2015 Feb 16;179(3):378–91. doi: 10.1111/cei.12434 (PMC4337671; doi:10.1111/cei.12434)

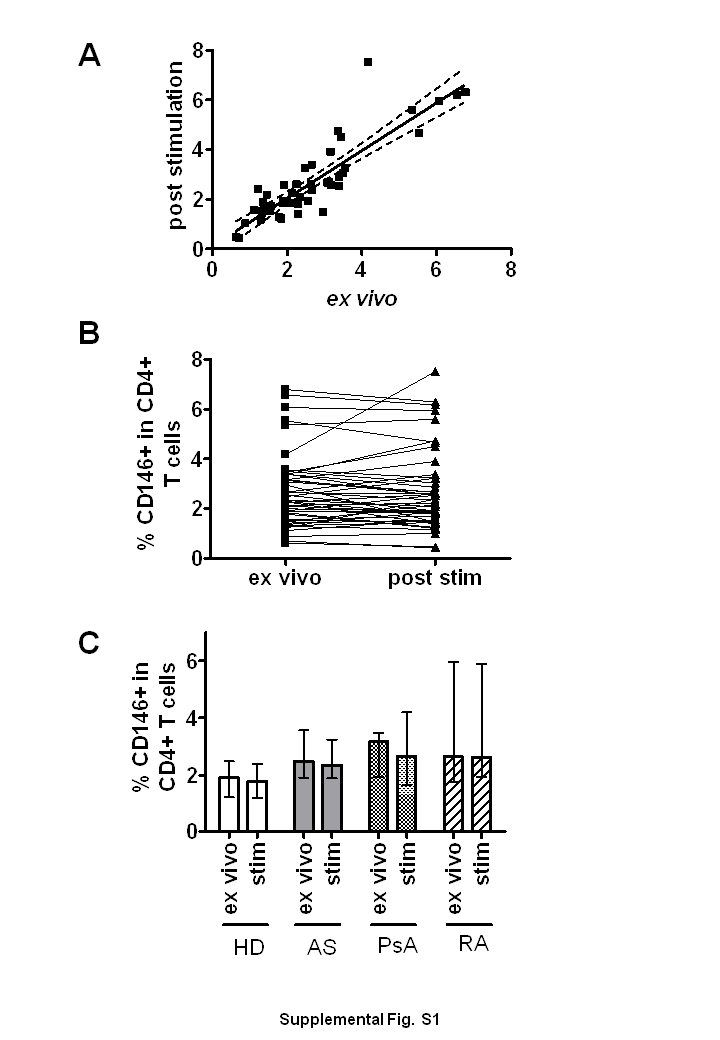

Supplement: Supplementary file 1 [file cei0179-0378-sd1.zip › cei12434-supp-0001-figureS1.TIF]

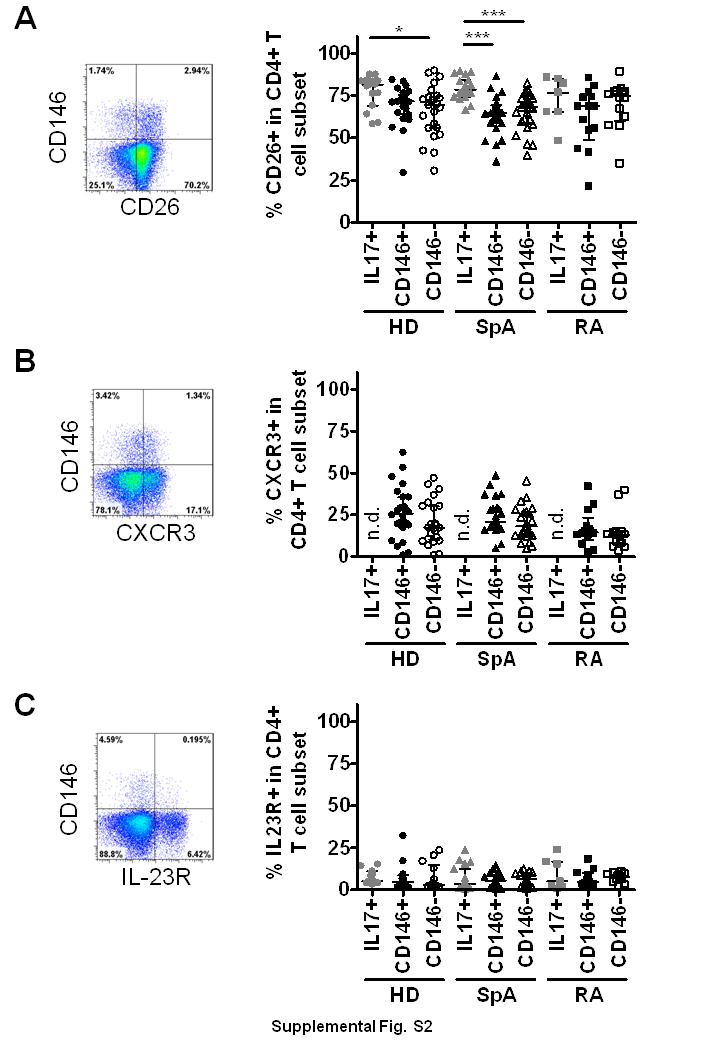

Supplement: Supplementary file 1 [file cei0179-0378-sd1.zip › cei12434-supp-0002-figureS2.TIF]

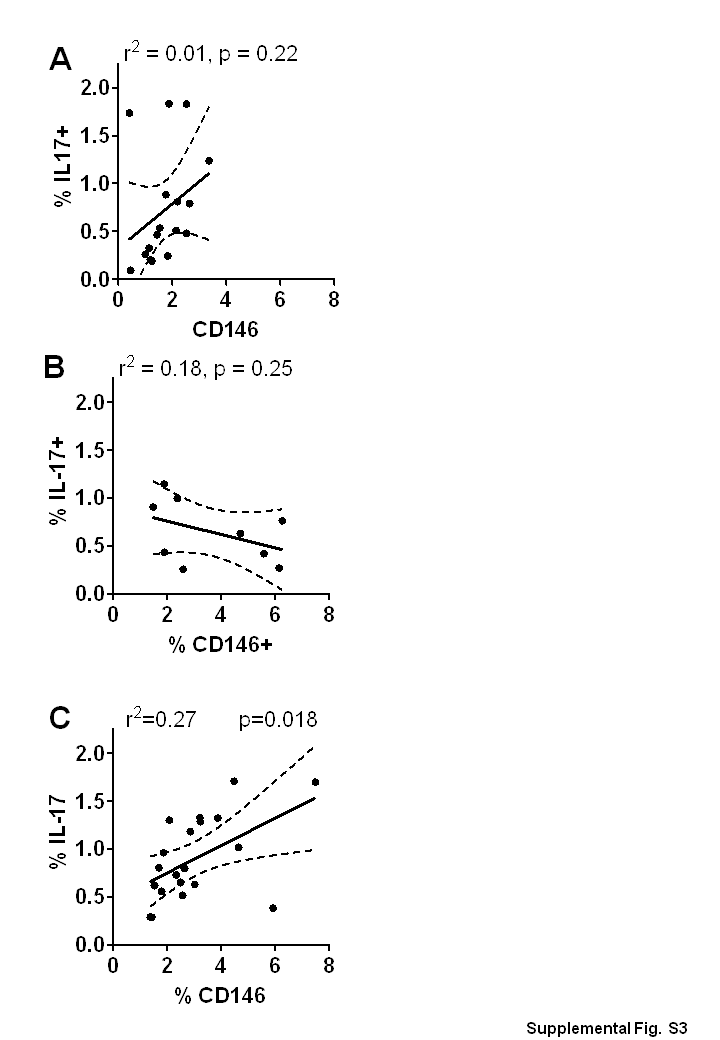

Supplement: Supplementary file 1 [file cei0179-0378-sd1.zip › cei12434-supp-0003-figureS3.TIF]

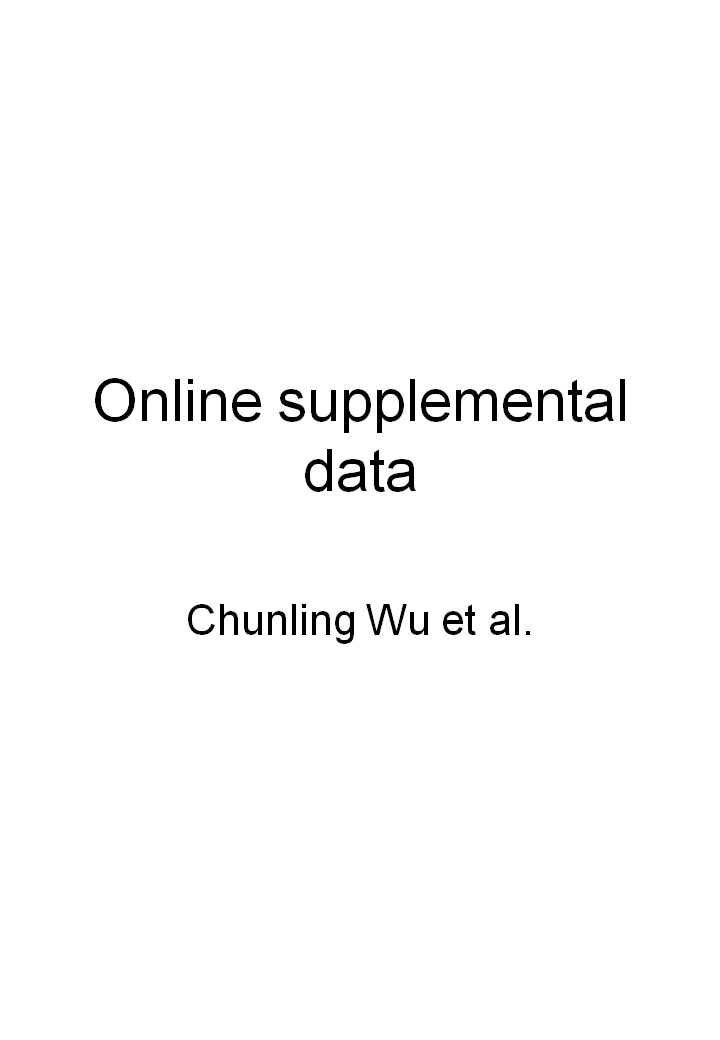

Supplement: Supplementary file 1 [file cei0179-0378-sd1.zip › cei_12434_online-supp-data.TIF]
